# George Forster Burder

**Published:** 1892-03

**Authors:** 


### Obituary.

### GEORGE FORSTER BURDER, M.D., F.R.C.P.

It is with much regret that we record the death of Dr. Burder, which took place very suddenly and unexpectedly on Saturday evening, February 6th. He had been out daily, as usual, and on the previous Thursday had read before the Bristol Naturalists' Society a very interesting paper on "Rainfalls and Floods in the neighbourhood of Bristol," and joined with much ability in the subsequent discussion as to the best mode of preventing these floods. But although he did not appear to be an invalid, Dr. Burder had, for some years, experienced at intervals feelings of angina and dyspnœa when walking uphill, or using more than ordinary exertion. On the evening of his death, after dining with some scientific friends, he walked quietly home, a distance of about half-a-mile, and immediately after entering his house complained of faintness and exhaustion, sat down in a chair, and expired, apparently without any pain or struggle. His death was quite in harmony with the even tenor of his life. On that Saturday evening, after his life's work was done, he sank quietly to his eternal rest.

At the time of his death, Dr. Burder was in his 68th year, having practised during the greater part of his life in this city, and held for many years important medical appointments. For instance, he was for twenty-seven years Physician to the Bristol General Hospital; viz., from 1856 to 1883, when he resigned, and was elected on the honorary and consulting staff. He was for twenty-three years lecturer on Materia Medica, and for nearly seventeen years Honorary Secretary, at the Bristol Medical School. Dr. Burder was also for many years a member of the Bath and Bristol Branch of the British Medical Association, and of the Bristol Medico-Chirurgical Society. He was elected President of the former Society in 1887, and of the latter in 1884. Members of the latter will not readily forget the masterly address which he then gave on the "Etiology of Catarrh," illustrated as it was by meteorological observations, made by him during a long series of years. To show that Dr. Burder was considered an authority on Meteorology many years ago, it may be mentioned that when the British Medical Association held its Annual Meeting in Clifton in 1863, Dr. Symonds being President, Dr. Burder was unanimously requested to read a paper on the Climatology of the District, and very ably he performed his task.

Dr. Burder was a Fellow of the Royal Meteorological Society, and of a similar Society in Scotland, and a member of the Bristol Naturalists' and Bristol Microscopical Societies — in fact his knowledge in other branches of Natural Science was but little inferior to his knowledge of Meteorology. Dr. Burder's death, like

Dr. Beddoe's retirement last year from Clifton and from medical practice here, leaves a gap amongst us which it is difficult to fill. They were both accomplished physicians. In a science, however, like medicine, by which men earn their daily bread, Nature abhors a vacuum, and eager aspirants soon rush in from all quarters to fill the void ; but it is not so in sciences such as Anthropology and Meteorology, which men cultivate more from natural inclination than from any immediate prospect of pecuniary reward—for love, in short, rather than for money. Dr. Burder never had a very large or lucrative practice. Money-making was evidently not his forte. Modest and unassuming, he did not understand keeping well to the front, or being always in evidence, and impressing people with smart, showy, but superficial qualities, which too often pass current with the public as profound learning. But those who had the discrimination to consult him as a physician never had cause to repent having taken this step. They found in him a man who thoroughly understood the use of remedies, who had sound judgment and excellent common sense, which has been well defined to be "genius in its working dress." In him also they found a man of genial and kindly disposition, of strict honour and integrity, and of ready sympathy with others. He was one who, in the course of his life, gained many friends; but, as far as we know, never turned a temporary opponent into an enemy.

"Multis ille bonis flebilis occidit."

